# Characterizing changes to harm reduction site operations in British Columbia following the implementation of the decriminalization of drugs: Findings from a provincial survey

**DOI:** 10.1186/s12954-025-01276-y

**Published:** 2025-07-18

**Authors:** Farihah Ali, Cayley Russell, Justine Law, Sameer Imtiaz, Juls Budau, Rita Shahin, Laura MacKinnon, Dylan Griffith, Sean Patenaude, Jessica Xavier, Jürgen Rehm

**Affiliations:** 1 Centre for Addiction and Mental Health (CAMH), Institute for Mental Health Policy Research, 250 College St., Toronto, ON M5T 1R8 Canada; 2 Ontario Node, Canadian Research Initiative in Substance Matters (CRISM), 250 College St., Toronto, ON M5T 1R8 Canada; 3 Faculty of Applied Health and Community Studies, Sheridan College, 7899 McLaughlin Road, ON L6Y 5H9 Brampton, Canada; 4 Dalla Lana School of Public Health, University of Toronto, 155 College Street, Toronto, ON M5T 3M6 Canada; 5 School of Population and Public Health, University of British Columbia, East Mall, Vancouver, BC 2206, V6T 1Z3 Canada; 6 Toronto Public Health, 277 Victoria St, Toronto, ON M5B 1W2 Canada; 7 Department of Family Medicine, University of British Columbia, Vancouver, BC V6T 2A1 Canada; 8 Kootenay Insurrection for Safe Supply, 40-622 Front St, Nelson, BC V1L 4B7 Canada; 9 Centre for Addiction and Mental Health, QuEST Team, 33 Ursula Frank St, Toronto, ON M5S 2S1 Canada; 10 BC Centre for Disease Control, 655 W 12th Ave, Vancouver, BC V5Z 4R4 Canada; 11 Department of Psychiatry, Dalla Lana School of Public Health, & Institute of Medical Science (IMS), University of Toronto, 1 King’s College Circle, Toronto, ON M5S 1A8 Canada; 12 Campbell Family Mental Health Research Institute, Centre for Addiction and Mental Health (CAMH), 1001 Queen St. West, Toronto, ON M6J 1H4 Canada; 13 Institute of Medical Science, Faculty of Medicine, University of Toronto, 6 Queens Park Crescent, ON M5S 3H2 Toronto, Canada; 14 WHO European Region Collaborating Centre, Public Health Institute of Catalonia, 81-95 Roc Boronat St, Barcelona, 8005 Spain; 15 Center for Interdisciplinary Addiction Research (ZIS), Department of Psychiatry and Psychotherapy, University Medical Center Hamburg-Eppendorf (UKE), Martinistraße 52, 20246 Hamburg, Germany

**Keywords:** Decriminalization, Drug policy, Harm reduction, People who use drugs, Public health

## Abstract

**Background:**

In January 2023, British Columbia (BC), Canada, piloted a three-year decriminalization policy to address the escalating overdose crisis. The policy seeks to reduce stigma and the fear of criminal prosecution, and foster a safer and more supportive environment, encouraging greater utilization of treatment and harm reduction (HR) services among people who use drugs. There are limited data on the operational characteristics of HR sites in BC, which are essential for monitoring how decriminalization may influence service operations and utilization. This study aimed to characterize HR site operations in BC and assess any operational changes following decriminalization.

**Methods:**

A cross-sectional, online self-report survey was distributed to HR sites across BC between March and April 2024. The survey was completed by a site representative, and survey questions focused on client demographics and drug use patterns, service uptake and capacity, resource and staffing demands, police activity near sites, and the availability of HR services. Changes pre-and post-decriminalization were analyzed descriptively to identify trends.

**Results:**

A total of 33 HR sites completed the survey. Almost a third (30%) of sites reported an increase in client’s post-decriminalization, and 18% indicated plans to expand or modify services to meet the increasing demand. However, challenges related to staffing and resources were highlighted, with 45% of sites reporting increased staffing demands post-decriminalization, and 33% noting changes to resource needs, most of which increased. Five sites reported an increase in annual operating budgets. Nearly half (43%) of sites that experienced police activity around their site reported increased police activity post-decriminalization. Moreover, approximately one-fifth (21%) of sites received formal decriminalization training.

**Conclusions:**

HR sites have experienced an increase in client engagement post-decriminalization, reporting challenges related to site capacity and funding, and emphasizing the need for additional investments to support and expand HR services. Consideration should be given to needs-based planning and providing decriminalization training to HR staff. Moreover, steps are necessary to address the continued police presence near sites, which may hinder service uptake and perpetuate stigma. Addressing these gaps is critical for improving health system engagement for people who use drugs and achieving the goals of decriminalization.

## Introduction

Harm reduction (HR) is a globally recognized public health strategy aimed at minimizing the negative consequences associated with substance use [1, 2]. As an integral part of the substance use system of care, HR operates on the principle that individuals who use drugs have the right to do so, challenging the notion that substance use is inherently harmful, morally wrong, or should be criminalized [1, 2]. Instead of focusing solely on reducing or eliminating substance use, HR emphasizes approaches, tools, and services designed to mitigate the physical, psychological, and social harms associated with use. This adaptive, person-centered approach benefits both people who use drugs (PWUD) and the broader community [1–7]. 

In Canada, several HR initiatives have been bolstered over the last several years. For instance, prescribed alternatives to illegal drug programs (also known as prescribed safer supply programs), which allow PWUD at a high risk of overdose to access pharmaceutical-grade medications (including select opioids, stimulants or benzodiazepines), have been introduced in several Canadian provinces as an alternative to the toxic illicit drug supply [1, 2, 8–10]. Other HR programs include supervised consumption sites (SCS) and overdose prevention sites (OPS), which provide safe spaces for PWUD to use pre-obtained drugs under the supervision of staff who are trained in overdose response (who are often people with lived or living experience with drug use, otherwise known as peers), thereby reducing the risks of overdose and disease transmission [11]. SCS are more permanent sites that operate legally through federal exemptions, while OPS are lower- barrier sites that only require the approval of the local medical health officer, as part of a legal designation [11]. Research has shown that both SCS and OPS increase opportunities for early intervention in overdose response, reduce drug use-related infections, improve access to health services, and subsequently decrease the risk of fatal and non-fatal overdoses [3, 4, 12]. In fact, according to Health Canada, no fatal overdose has ever occurred in a SCS in Canada [13]. Importantly, while available evidence on OPS is limited, the programs that were evaluated have not been associated with increased drug use or drug-related crime and are considered cost-effective, with savings in medical care offsetting operational costs [14, 15]. 

In addition, other health services for PWUD that emphasize HR principles, such as Rapid Access Addiction Medicine (RAAM) or Rapid Access Addiction Clinics (RAAC), as well as Mental Health and Substance Use (MHSU) sites, have been increasingly implemented across the country [16, 17]. These clinics offer a range of services, including addiction assessment, counselling, access to addiction specialists, medication-assisted treatment (e.g. Opioid Agonist Treatment [OAT]), and linkages to broader community supports, without requiring appointments or referrals [18]. Key components of HR also include providing equipment for safer injecting and smoking to prevent the transmission of infections such as HIV and hepatitis C, as well as ensuring the safe disposal of used equipment [1, 2]. Additional measures include the distribution of take-home naloxone kits to reverse opioid overdoses, access to opioid replacement medications, drug-checking services, drug use and overdose education, counseling, and referrals to treatment and other support services [1, 2]. 

The Canadian province of British Columbia (BC) has a long history of pioneering HR initiatives. Notably, BC was the first jurisdiction in North America to grant an operating permit to open a SCS, opening Insite in 2003 [19]. BC’s leadership in HR can be attributed to a sustained history of public health advocacy led by researchers, healthcare professionals, and grassroots organizations of PWUD, alongside the province’s response to the overdose crisis [19]. Currently, HR services are a foundational component of BC’s substance use care system, and are primarily funded by the provincial government through its regional health authorities [20]. Service delivery is typically managed directly by health authorities or through partnerships with non-profit and community organizations, allowing for the regional adaptation of services to meet local needs.

Despite its longstanding leadership in HR, BC has remained the epicenter of Canada’s overdose crisis, with overdose deaths nearly tripling between 2019 and 2023, rising from 990 to 2,558 [21–23]. In 2023, fentanyl and benzodiazepines were detected in 85% and 40% of toxic drug deaths, respectively, while cocaine and methamphetamine were detected in 49% and 38% of toxic drug deaths, underscoring the polysubstance nature of the unregulated drug supply [24]. This pattern is largely driven by the extreme volatility of the unregulated market, which continues to be dominated by illicitly manufactured fentanyl, fentanyl analogues, and other synthetic opioids such as nitazenes, alongside a growing presence of novel benzodiazepines [25]. However, a range of contextual and structural factors have further amplified the drug toxicity crisis in BC. These include high rates of poverty, persistent housing insecurity, and inequitable access to health and HR services across regions [26]. Compared to many international jurisdictions, where fentanyl has only recently begun to enter drug markets and where more robust social supports are in place, BC has faced a prolonged and severe overdose crisis spanning decades [27, 28]. Despite the urgent need for HR services to mitigate these harms, significant barriers persist, including stigma and the criminalization of drug use, which have been found to contribute to a lack of engagement with HR services across the province [29–35]. 

To address longstanding issues around drug use and service accessibility, BC introduced a three-year initiative in 2023 to decriminalize the personal possession of small amounts of certain drugs for adults (18+) [36]. Under this policy, adults carrying a cumulative total of 2.5 g or less of opioids, methamphetamine, cocaine/crack-cocaine, and MDMA/ecstasy are no longer subject to arrests or drug confiscation [36]. Beyond reducing drug-related arrests, the policy has several longer-term goals, such as reducing stigma associated with drug use, and increasing awareness, engagement, and utilization of services province-wide, including HR programs. Ultimately, the increased accessibility of these programs is proposed to mitigate the harms associated with drug use, including overdoses [36–38]. Realizing this potential will require a comprehensive understanding of the current HR landscape in BC, including an assessment of how the policy will impact access to and utilization of HR services by PWUD across the province. Ongoing monitoring and evaluation of the policy’s implementation and outcomes will be essential to ensure its effectiveness, and guide necessary adjustments to meet its goals.

There are limited data available on the operational aspects of HR sites in BC, including their hours of operation, range of services offered, staffing structures, and funding models. Understanding these operational details is critical, as they provide insights into how HR services may have adapted in response to decriminalization. While these operational indicators are not explicit goals of the policy, they are essential for assessing access to, and utilization of, HR services, and for evaluating whether sites have evolved to meet the changing needs of PWUD. To address this gap, our study examined the operational characteristics of HR sites in BC, and assessed whether any operational changes occurred following the implementation of decriminalization. Specifically, we explored factors such as HR site uptake, capacity, demand, client demographics and drug use profiles, resource and staffing demands, and police activity near sites. These data can help illuminate whether and how HR sites have altered their operations in response to the policy, and provide important context for understanding service accessibility and responsiveness for PWUD. The findings presented reflect the first year of a multi-year longitudinal mixed-methods evaluation of the impact of decriminalization on HR site operations in BC, and will provide valuable in sights to guide future policy and practice in the province.

## Methods

### Study Design

We conducted a cross-sectional survey of HR site operations using an online, self-administered survey distributed to site representatives who had operational knowledge of the site (such as directors, program managers, or supervisors).

### Eligibility

Eligible HR sites included SCS, OPS, RAAM or RAAC clinics, MHSU sites, and low-barrier sites offering drug checking, OAT, needle exchange/disposal and mobile outreach. Shelters and supportive or temporary housing settings that housed registered OPS were also included. However, broader community health centers/clinics, public health units, and pharmacies that distributed HR supplies were excluded, as their primary mandate was not addiction-focused. Additionally, sites that exclusively served youth were excluded as the decriminalization policy applies specifically to adults (aged 18+). Sites that opened after the implementation of decriminalization were also excluded as they were not able to comment on the impacts of decriminalization.

### Recruitment

We undertook a multi-pronged recruitment strategy to identify both eligible HR sites, as well as a representative from each eligible HR site to respond to the survey on the site’s behalf. First, we compiled an initial list of HR sites across BC using established online directories, including Toward the Heart, and the BC Centre for Disease Control (BCCDC) harm reduction service website [39]. This preliminary list was then organized by BC’s five regional health authorities: Fraser Health, Vancouver Coastal Health, Interior Health, Island Health, and Northern Health. We then shared the draft site list with the project’s working group, which included people with lived and living experience with drug use, as well as with BCCDC’s Harm Reduction Coordinator team and the Decriminalization Leads from each of BC’s health authority (i.e., staff designated to support the implementation of BC’s decriminalization policy). This collaborative review process served multiple purposes. First, it allowed for validation and refinement of the site list, enabling the identification of eligible sites we may have missed and the removal of those that did not meet our inclusion criteria. Second, it helped us identify appropriate site representatives who were best positioned to respond to the survey. For sites where no direct contact information was provided, our partners disseminated the survey via email through their networks. We also conducted supplementary outreach through online searches and telephone calls to independently identify appropriate contacts. All outreach materials clearly stated that the survey should be completed by someone with sufficient knowledge of site operations, staffing, and service delivery. However, no strict eligibility criteria was imposed regarding the respondent’s role or title.

### Measures

The survey questions were co-designed with the project’s working group and included both multiple-choice and open-ended qualitative questions, focusing on key areas of HR service operations, including service capacity, services offered, service utilization/uptake, available resources (personnel, infrastructure, and funding), referral pathways, client demographics and substance use profiles. For each measure, follow-up questions were included asking whether it had changed since decriminalization (for instance, “since decriminalization (after January 31, 2023), have your operating days/hours changed?”). Given that this line of questioning makes it difficult to know whether the changes were explicitly attributed to the decriminalization policy or were instead temporally associated, to account for this, when participants indicated yes, we asked an additional open-ended follow up question asking them to expand on the impacts to provide additional context. This approach allowed us to capture both the current landscape of HR services and any changes in service delivery and operations since the implementation of decriminalization, as well as assess the extent to which the policy influenced these changes.

### Data Collection

The final survey was built using Research Electronic Data Capture (REDCap) software, a secure web application for building and managing online surveys and databases [40]. Survey invitations were sent out on a rolling basis between March 15 and April 10, 2024. Our contacts with the BCCDC and the Decriminalization Leads from each Health Authority directly emailed site representatives they had identified, while all remaining sites were directly emailed by our project team using REDCap’s automatic email function. Each identified representative received an email inviting them to participate in the study, that also included a link to an informed consent form they were required to sign to complete the survey. To boost participation, we set the survey fielding period to two months in advance and planned to send two reminder emails at two-week intervals following the initial invitation. Data collection concluded on May 31, 2024. All participants were offered an optional $25 Amazon e-gift card upon survey completion.

### Data Analysis

Data were exported from REDCap into Excel and were analyzed descriptively (e.g., frequency distributions and percentages for each measure). Data were categorized and presented under the following three major headings: HR Site Operations and Characteristics, Clientele Profiles, and Police Activity around the Site. We also classified the community each HR site was located in as rural or urban using Statistics Canada’s Index of Remoteness (RI), which assigns a score between zero (very accessible, not remote), to one (not accessible, very remote) based on the community’s population size and distance to population centres [41, 42]. Communities with an RI score below 0.29 were classified as rural, while those with scores above 0.29 were considered urban [42]. 

### Ethics

This study was reviewed and approved by the Centre for Addiction and Mental Health research ethics board (REB#2023/169).

## Results

A total of 142 HR sites were invited to participate in the survey. Six sites were excluded (*n* = 2 were youth-specific and did not meet the eligibility criteria, *n* = 2 were duplicate responses from the same sites, and *n* = 2 had opened post-decriminalization and could not provide feedback on its impact). After these exclusions, *n* = 33 sites completed the survey, representing a 23% response rate.

Site representatives who completed the survey included program managers (*n* = 8), supervisors (*n* = 8), directors (*n* = 4), support workers (*n* = 3), and nursing staff (*n* = 3). Other respondent roles included a program coordinator, clinician, board chair, administrative lead, case manager, Indigenous patient navigator, and crisis counselor. The median length of time participants reported working in their current roles was 4.5 years (interquartile range [IQR]: 2.6).

### HR Site Operations and Characteristics

#### HR Site Location

The 33 sites surveyed were located across all five of BC’s health authority regions. Specifically, more than a third (*n* = 12/33; 36%) of sites were located in the Island Health Authority, followed by nine (*n* = 9/33; 27%) in the Interior Health Authority, six (*n* = 6/33; 18%) in the Vancouver Coastal Health Authority, four (*n* = 4/33; 12%) in the Fraser Health Authority, and two (*n* = 2/33; 6%) in the Northern Health Authority.

**Fig. 1:**
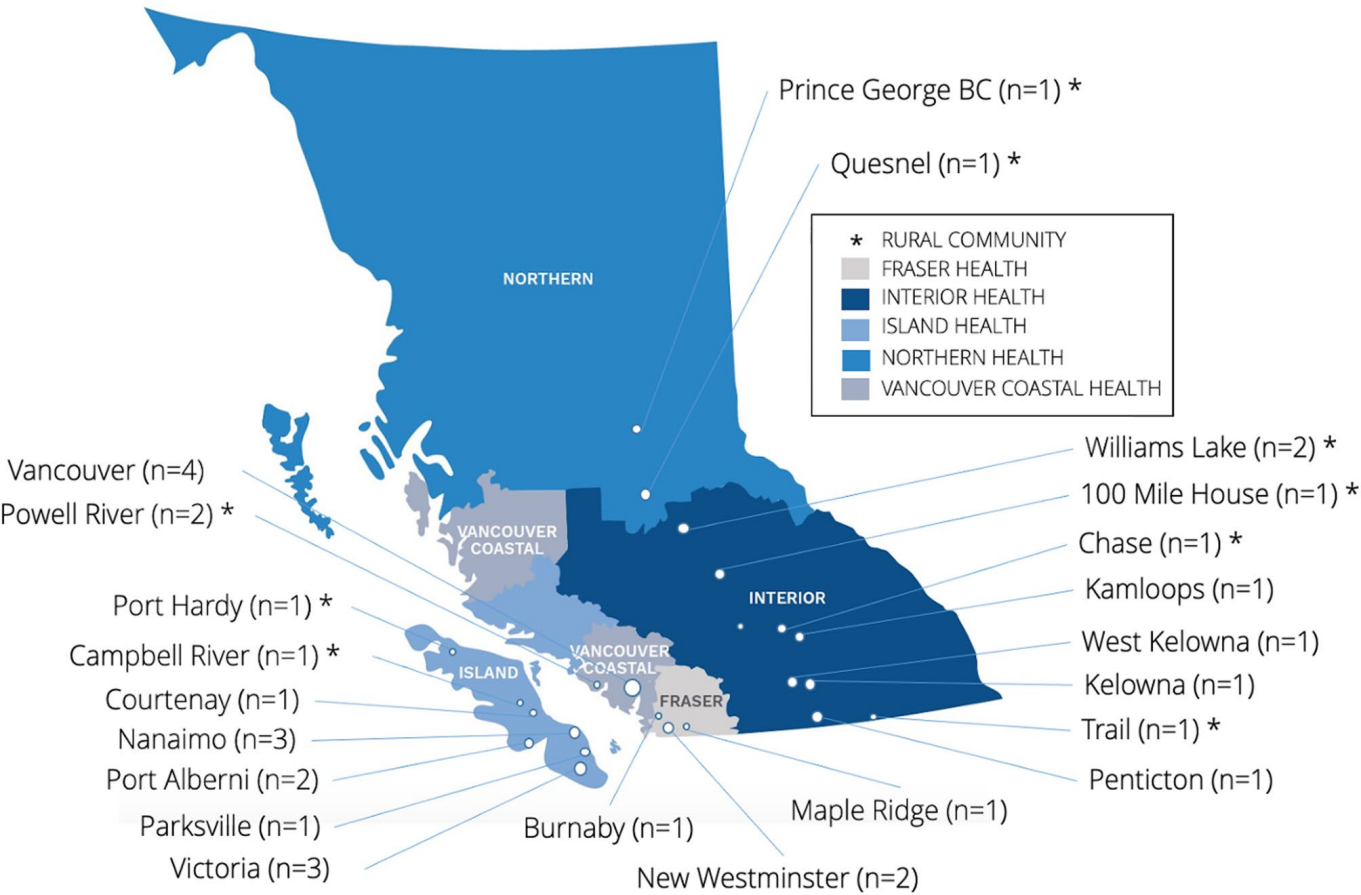
Map of HR site survey respondents by community and BC health authority region. (Adapted from HealthMatch BC) [43]

Over two-thirds (*n* = 22/33; 67%) of the HR sites were located in urban areas, ten (*n* = 10/22; 45%) of which were located in the Island Health Authority region, four (*n* = 4/22; 18%) were in the Fraser Health Authority region, four (*n* = 4/22; 18%) in the Interior Health Authority, and four (*n* = 4/22; 18%) in Vancouver Coastal Health Authority region. The remaining eleven sites (*n* = 11/33; 33%) were located in remote or rural communities in the Interior Health Authority (*n* = 5/11; 45%), Island Health Authority (*n* = 2/11; 18%), Vancouver Coastal Health Authority (*n* = 2/11; 18%), and the Northern Health Authority (*n* = 2/11; 18%). See Fig. 1 for a map of HR site locations by community and BC health authority, with reference to the rurality of cities.

#### Site Establishment and Infrastructure

Among the 22 h sites that provided information on their establishment date, five (*n* = 5/22; 23%) had been operating for over 20 years, with the earliest opening in 1999. More than half (*n* = 12/22; 54%) of sites were established between five to 20 years ago, while the remaining five sites (*n* = 5/22; 23%) were established within the last five years.

Regarding site infrastructure, 13 sites (*n* = 13/33; 39%), were fully integrated within a broader organization that offered additional health or social services, and ten (*n* = 10/33; 30%) were affiliated with a broader organization but operated at a separate location. One site was uncertain about its affiliation status.

#### Site Hours of Operation

Nearly half (*n* = 16/33; 48%) of HR sites reported being open seven days a week, while an additional 15 (*n* = 15/33; 45%) were open five days a week. One site operated six days a week, and another was open four days a week. In terms of weekend operations, over half (*n* = 18/33; 54%) of sites indicated that they were open on Saturdays, while a similar proportion (*n* = 17/33; 52%) of sites were open on Sundays. Sites operated for an average of 9.43 h per day (SD: 0.3), with opening hours ranging anywhere from 7am to 6pm, and closing hours ranging from 2pm to 2am. Nine sites (*n* = 9/33; 27%) indicated that they were open past 6pm, while one site reported that they operate 24 h a day, seven days a week. Another site opened both during the day (from 9 am to 6 pm), and at night (from 7 pm to 11 pm), with a period of closure in between.

To understand service operations, survey respondents were asked if their HR site made any changes to their hours of operation following the implementation of the decriminalization policy, the majority (29/33; 88%) of which had not. However, two sites reported increasing their hours of operation, while another two indicated that they decreased their hours of operation since decriminalization.

#### Services Offered

HR sites offered a wide range of services. All sites reported offering HR supplies (e.g. sterile drug use equipment and naloxone) and training, while the majority (*n* = 24/33; 73%) provided overdose prevention services, followed by peer support (*n* = 23/33; 70%), and drug checking services (*n* = 22/33; 67%). Additional services offered across sites are outlined in Table 1.

**Table 1:** HR services offered by sites (*n* = 33)

Service	Total (*n* = 33)	
	*N*	%
Harm reduction supplies distribution (including naloxone)	33	100
Overdose prevention services*	24	73
Peer support	23	70
Drug checking	22	67
Mental health counselling	18	54
Substance use counselling	18	54
Mobile outreach	18	54
OAT prescriptions	17	52
Clinical/Wound care	16	48
Health education	14	42
Supervised injection services**	14	42
Community syringe/paraphernalia pick up	13	39
Sexually transmitted/blood-borne infection testing and prevention	12	36
Prescribed alternatives to illegal drugs	11	33
Supervised inhalation services**	8	24
Withdrawal management	8	24
Social and family support	7	21
Indigenous-specific services	5	15

* Overdose prevention services were not necessarily sites that were registered as OPS; rather, these reflected sites that provided any type of overdose prevention

**This is not reflective of whether the sites were registered as OPS/SCS but is rather reflective of the mode of consumption permitted at the site

Over three-quarters (*n* = 25/33; 76%) of sites indicated that they did not introduce any new services directly in response to the decriminalization policy. However, one noted that they had started offering workshops and resources to educate individuals on the decriminalization policy and its goals. Moreover, six sites (*n *= 6/33; 18%) indicated plans to expand or modify their services in the near future based on the decriminalization policy. Planned changes proposed by sites included implementing supervised inhalation services, expanding dispensing machine options for HR supplies, and introducing a clean-up program to address drug-related paraphernalia and litter around the site. One site indicated that they plan to relocate to a larger building to increase service capacity, while two sites expressed intentions to expand their services to meet the growing demands post-decriminalization, though they did not specify which areas or services they intended to focus on for the expansion.

#### Clientele Focus

Five sites (*n* = 5/33; 15%) reported that their site had a specific clientele focus. One served clients with concurrent mental health and addiction disorders, one was a women-only site, and the remaining three focused on LGBTQ2S + individuals, Indigenous communities, and people who inject drugs, respectively. Two sites indicated they served site-specific clients (e.g., existing inpatient/outpatient clients or those formally involved in a democratic-run member organization of PWUD). Data on whether sites’ clientele focus had changed since the implementation of decriminalization were not collected.

#### Site Uptake

HR site respondents also reported the average number of clients visiting their site each month. Most sites (*n* = 17/33; 52%) indicated that they serve over 200 clients monthly, while nine (*n* = 9/33; 27%) reported serving between 25 and 100 clients per month. Three additional sites (*n* = 3/33; 9%) served between 101 and 200 clients every month, while four sites (*n* = 4/33; 12%) were unsure of their average monthly clientele.

Nearly a third of all HR sites (*n* = 10/33; 30%) reported an increase in the number of clients accessing their services after decriminalization. Eight sites (*n* = 8/33; 24%) were not certain whether their site had seen a change in the number of clients accessing their service.

#### Site Demand

The majority (*n* = 23/33; 70%) of sites reported having to turn away clients or ask them to wait in a waiting room or lineup due to insufficient capacity or resources. Data on whether the waitlists had changed since the implementation of decriminalization were not collected.

#### Funding and Operating Budgets

HR sites reported receiving full or partial funding from a combination of public and private sources. The majority (*n* = 26/33; 79%) of sites received provincial funding. Three sites (*n* = 3/33; 9%) were municipally funded, and one site was federally funded. An additional five sites (*n* = 5/33;15%) received funding through donations or fundraising efforts. Other funding sources included research grants, universities, and non-profit organizations.

None of the sites indicated their funding source had changed since decriminalization. One site commented that there had been an increase in funding opportunities due to the site’s growing credibility and capacity, but that their funding was still insufficient to meet service demands.

Annual site operating budgets varied substantially across sites. Among the 16 sites (*n* = 16/33; 48%) that were aware of their organization’s annual operating budget, six (*n* = 6/16; 38%) reported annual budgets between $500,000 and $1 million, while four (*n* = 4/16; 25%) had budgets ranging between $250,000 and $500,000. Three sites (*n* = 3/16; 19%) had budgets between $100,000 and $250,000, while another three reported budgets exceeding $1 million. The remaining 17 sites (*n* = 17/33; 51%) were unsure of their annual site operating budgets. Following decriminalization, five sites (*n* = 5/16; 31%) reported that their total operating budget had increased.

#### Staffing and Resources

Site respondents provided information on the type of staff currently employed at their HR site. Over one third (*n* = 13/33; 39%) of sites reported being staffed by multidisciplinary teams consisting of administrative personnel (including intake workers), managers, medical practitioners (either registered nurses, nurse practitioners, or physicians), peer support providers (e.g. peers/people with lived experience, community workers, outreach workers, and frontline or overdose response workers), and behavioural or social support staff (e.g. social workers, and counsellors). The remaining sites had various combinations of these roles. Table 2 provides a breakdown of the number of HR sites that employed staff in each employee role.

**Table 2:** Harm reduction site employee roles (*n* = 33)

Employee Roles	Total (*n* = 33)	
	*n*	%
Peer support providers	30	91
Managers	23	70
Medical Practitioners	20	61
Administrative personnel	20	61
Behavioural or social support workers	17	52

The majority (*n* = 17/33; 52%) of sites reported that their current staffing levels were insufficient to meet client demand, while three sites (*n* = 3/33; 9%) were unsure whether their current staffing met their clients’ needs. Following decriminalization, nearly half (*n* = 15/33; 45%) of all sites reported an increase in staffing demands.

Regarding site resources, two-thirds (*n* = 22/33; 67%) of sites reported that their current resources were insufficient to meet client demands, with one site unsure. One-third (*n* = 11/33; 33%) of sites experienced changes in resource demand following decriminalization, with increases experienced across the majority of resource demand categories. See Table 3 for an overview of the perceived impacts of decriminalization on staffing and resource demand across HR sites.

**Table 3:** Perceived impacts of decriminalization on staffing and resource demand Post-Decriminalization

Perceived Impacts of Decriminalization on Staffing Demand	Total (*n* = 15)	
	*n*	%
***Workload***	**12**	**80**
Increased	12	80
***Community outreach***	**11**	**73**
Increased	11	73
***Administrative tasks***	**10**	**67**
Increased	10	67
***Training and development***	**10**	**67**
Increased	10	67
***Client interactions***	**10**	**67**
Increased	10	67
***Client intake***	**8**	**53**
Increased	8	53
***Overdose prevention/response***	**7**	**47**
Increased	6	40
I don’t know	1	7
***Scheduling***	**4**	**27**
Increased	3	23
I don’t know	1	7
**Perceived Impacts of Decriminalization on Resource Demand**	**Total (*n*** = **11)**	
***Medical/harm reduction supplies***	**9**	**82**
Increased	8	73
Decreased	1	9
***Space/facility usage***	**6**	**54**
Increased	5	45
I don’t know	1	9
***Educational materials***	**6**	**54**
Increased	5	45
Decreased	1	9
***Technological equipment***	**4**	**36**
Increased	4	36
***Transportation***	**4**	**36**
Increased	3	27
I don’t know	1	9

#### Decriminalization Training

Seven sites (*n* = 7/33; 21%) reported that their staff had received formal decriminalization training, while five (5/33; 15%) were unsure. Training formats varied. Six sites (*n* = 6/7; 86%) completed the training virtually through online modules or presentations/meetings with health authority-employed decriminalization leads, while one site received training via emails and resources from their health authority and drug user advocacy groups. Training duration ranged from one hour to one full day. Nearly three-fourths (*n* = 5/7; 71%) of all sites that received training felt it provided staff with adequate knowledge of the decriminalization policy. Among the remaining 21 sites that did not receive decriminalization training, most (*n* = 13/21; 62%) expressed that their staff would benefit from such training.

### Clientele Characteristics

Our study also examined clientele characteristics at HR sites, including demographics (age, gender, and ethnicity) and substance use practices (drugs of choice and routes of administration), based on site observations, as well as whether these characteristics had changed since decriminalization.

#### Demographics

The majority (*n* = 27/33; 82%) of sites served clients of all ages, primarily those above age 18. Three sites (*n* = 3/33; 9%) reported that their primary clientele was between 30 and 49 years old, while one additional site primarily served younger clients aged 18 to 29. Two sites were uncertain about the age of their primary clientele.

Additionally, nearly half (*n* = 16/33; 48%) of sites reported serving an equal number of male-identifying, female-identifying, and gender-expansive clients, while four (*n* = 4/33; 12%) served an equal number of men and women, but fewer gender-expansive clients. Men were the primary clientele at five sites (*n* = 5/33; 15%), and an additional two sites indicated that they primarily served gender-expansive clients. Six sites (*n* = 6/33; 18%) indicated that they did not know the gender of their primary clientele.

In terms of racial demographics, white individuals were the primary clientele at the majority (*n* = 23/33; 70%) of sites, while Indigenous individuals made up the majority of clients at eight sites (*n* = 8/33; 24%). Two sites were uncertain of the most common ethnicity of their clientele.

Three sites (*n* = 3/33; 9%) indicated changes in clientele demographics post-decriminalization. One site noted serving a broader age range of clients, ranging from 16 to over 70 years old. Another site observed an increase in clients who identify as transgender, while a third site reported an increase in Indigenous clients accessing their services since decriminalization.

#### Substance Use Practices

Site respondents were asked to identify the top three illegal or non-prescribed substances most commonly used by clients at their site, along with the primary route of administration for each. Most sites (*n* = 20/33; 61%) reported that the most frequently used substance among their clients were illegal/street-sourced opioids. This was followed by methamphetamine (*n* = 10/33; 30%), and stimulant/opioid combinations (e.g. speedballs, goofballs) (*n* = 7/33; 21%). Inhalation was the primary route of administration reported for all three commonly used substances at sites.

Following decriminalization, three sites (*n* = 3/33; 9%) observed changes in the primary substances used by their clients, and three sites (*n* = 3/33; 9%) noticed an increase in the frequency of substance use among their clients. Four sites (*n* = 4/33; 12%), were unable to assess changes in the frequency of use, as they reported seeing different clients pre- versus post-decriminalization.

### Police Activity Around the Site

Nearly 70% (*n* = 23/33) of all sites reported experiencing police activity on or near their site’s premises, while two sites were unsure if police activity occurred near their locations. Among these sites, ten (*n* = 10/23; 43%) reported an increase in police activity following decriminalization. Conversely, two sites reported a decrease in police presence, while two sites reported they were uncertain about whether police activity had changed since decriminalization was implemented.

## Discussion

The findings from this study provide insights into the current landscape of HR sites across the province of BC, as well as early changes to site operations since the implementation of decriminalization. These data provide a baseline from which to monitor potential changes to HR operations going forward, and provide a snapshot of self-reported operational changes that have occurred since the implementation of the policy. The overall utilization of HR services demonstrates an upward trend, with nearly a third of surveyed sites reporting increased client volumes following decriminalization. This pattern aligns with data from the BC Ministry of Mental Health and Addictions that similarly indicate rising use of some HR services, such as drug-checking and take-home naloxone kits, since the policy came into effect [44]. However, it is challenging to fully assess the impact of decriminalization on these indicators due to potentially confounding factors, such as the housing crisis, which has been worsening across the province and may result in precariously housed individuals relying on low-barrier HR sites for basic necessities and support, as well as the volatility of the drug supply [25, 45, 46]. Regardless of whether the increased engagement can be specifically attributed to decriminalization, our findings reveal a growing strain on the ability for HR sites to meet this increasing demand. For instance, more than half of sites reported that their current staffing levels were inadequate, and two-thirds indicated that they have insufficient resources. The resource and staffing constraints are longstanding; nearly three-quarters of sites reported that they had to turn clients away or place them on waiting lists due to capacity constraints even before decriminalization, with sites indicating these issues have been aggravated since the implementation of the policy. An increase in demand for these health and social services could result in longer wait times, more clients being turned away, and a reduced capacity to provide comprehensive care - factors that may deter PWUD from seeking future support and ultimately undermine the intended benefits of decriminalization [47]. 

If sites were already operating at capacity pre-decriminalization, it is unlikely they will be able to expand services or introduce new ones post-decriminalization without additional funding support. The government’s decriminalization application explicitly outlined the need for adjunct investments in health and social services, including HR services to support the policy’s effectiveness. For instance, in tandem with the decriminalization policy, the government listed several initiatives with corresponding financial commitments, including a $22.6 million investment over three years (beginning in 2021) for prescribed pharmaceutical alternatives programs, and a recent (2024) allocation of $215 million over the next three years for mental health and addictions services [48, 49]. This latter investment includes $49 million to sustain existing HR initiatives such as SCS/OPS, drug-checking and naloxone kit distribution, along with $120 million earmarked for treatment programs [48, 50]. However, despite the increased demand, our data suggest that HR site funding has not been adjusted or increased to match the needs of a larger client base, increased staffing, and resource demands. Notably, none of the sites reported new funding sources following decriminalization, and only a small number of sites reported an increase in their annual operating budgets since decriminalization; the specific services the funding was allocated to is also unknown. This demonstrates the need to conduct needs assessments across HR sites in order to increase and target investments into the services that PWUD require, which can better support sites to meet clients’ needs [51, 52]. 

Given the reported increases in client visits post-decriminalization, nearly one-fifth of sites expressed plans to expand and/or modify their services to better accommodate clients. However, recent drug-related policy amendments in BC, which have largely been driven by a growing contempt among the public towards the decriminalization policy, may have hindered the ability of HR sites to expand services to meet clients’ needs [53]. For instance, sites that had planned to implement HR supply dispensing machines (which provide a low-barrier and cost-effective means of increasing access to HR supplies) are no longer able to do so as the BC government has suspended the operations of these machines, pending a review [54]. These machines reduced the service burden on pharmacies and HR programs, and minimized barriers to accessing HR supplies among PWUD, filling critical gaps in the overdose response system by reaching individuals who might not otherwise access HR or primary health services [55]. Moreover, in May 2024, the government also introduced new legislation that effectively re-criminalized the possession and use of drugs in public places. The amendment lists HR sites such as SCS/OPS, drug checking, places that provide outpatient addiction services, and places where people are legally sheltering as the only places aside from private houses where drug use and possession under the 2.5 g threshold is still legally permitted [37, 56]. This will likely further impact the growing demand on HR sites as PWUD who are experiencing homelessness or are unhoused will have nowhere else to legally use their drugs [57]. Alternatively, these policy changes may further entrench stigma against drug use and PWUD, discouraging them from accessing support, and ultimately undermining the long-term goals of the decriminalization policy [57]. 

In addition, there remains a notable gap in supervised inhalation services, with less than a quarter of sites providing this option to clients. These spaces provide a more hygienic and safe environment for PWUD to smoke their drugs under the supervision of trained staff and without fear of criminalization [58, 59]. The lack of these services is therefore a concerning shortfall, given that inhalation was reported as the primary route of administration for all commonly used drugs among clients in this survey, and has become the most common route of administration used in overdose deaths caused by illicit drugs in BC since 2017 [60]. This service gap requires targeted policies and investments to ensure access to SCS/OPS, and specifically supervised inhalation spaces. In the absence of sufficient spaces for safe and supervised inhalation, many people may have no choice but to continue consuming drugs in public, placing them at a greater risk of criminalization, subsequently countering the goals of the policy [57, 61]. 

The risk of criminalization remains a concern for PWUD, particularly in light of reported police presence near HR sites. Survey respondents indicated varying levels of police activity around their sites, with the majority reporting at least some police presence. Notably, nearly half of these sites observed an increase in police activity following decriminalization, while nine sites reported no change, two reported a decrease, and two were uncertain. These mixed findings likely reflect regional differences or urban-rural variation in how decriminalization is implemented and experienced on the ground. Perceptions of increased police presence may have serious implications for service uptake and accessibility. A visible police presence near HR sites can heighten fears of surveillance and criminalization, amplify stigma, and reinforce dominant societal narratives that frame drug use as a criminal issue rather than a public health concern [62–64]. These dynamics can deter PWUD from accessing HR services, thereby potentially increasing the risk of overdose and other health complications, such as those associated with unsafe inhalation practices [58]. They may also negatively influence public perceptions of drug policy reform and reduce community acceptance of HR sites [64–66]. To ensure alignment with the public health goals of decriminalization, efforts should be made to minimize visible police activity around HR service locations and to foster an environment that supports safe and non-punitive access to care [64–66]. 

Finally, additional initiatives are also crucial for fostering an environment that supports HR services and enhances the accessibility and utilization of these sites among PWUD. For instance, although only a small number of sites received decriminalization-specific training, there was strong interest among the remaining sites to receive this training. The limited number of sites receiving formal decriminalization training may have implications on the ability for sites to align their service provision with the goals of the policy. Such training could be invaluable, as it would equip staff with a comprehensive understanding of the nuances of decriminalization and its objectives, including how they can reinforce harm reduction principles and create a safe environment for open communication with clients who may be uncertain about their rights. This would enable them to effectively support PWUD under the new framework, and help bridge gaps in understanding between public health and the justice system [67–69]. Additionally, trained staff would be better prepared to address misconceptions when facing stigmatization from law enforcement, the public, as well as other healthcare personnel, toward PWUD [67–69]. As such, by aligning HR practices with decriminalization goals, sites can improve service delivery, contribute to the reduction of structural stigma, and promote trust, respect, and understanding between PWUD and service providers, ultimately increasing service engagement [67–69]. 

### Strengths and Limitations

This study included respondents from all five health authorities and from sites offering a wide range of HR services across BC. Missing data amounted to less than 0.7% across all measures, and almost two thirds (58%; 19/33) of sites indicated they systematically collect data on client sociodemographic information, improving the reliability of the responses to these questions. However, the total sample size and response rate were low, raising questions about the representativeness of the findings, and the comparability between sites that responded versus those that did not. It is possible that participating sites differed in distinct ways, for example, in terms of service capacity, geographic location, or operational challenges. Such differences may influence the findings and should be considered when interpreting the results. We also cannot be certain that every HR site in the province was identified and received the survey invitation, potentially limiting the generalizability of our results. Additionally, the diversity of respondent roles, which ranged from frontline staff (e.g., nurses, support workers) to managers and board chairs, may have influenced the specificity of responses. For instance, some respondents may have lacked access to or detailed knowledge of operational or budgetary information, while others may have been less familiar with day-to-day service delivery, potentially contributing to gaps, inaccuracies, or unknown responses.

Other limitations include the potential for biases inherent in self-reported data, such as response bias, recall bias, social desirability bias, and interpretation bias, all of which may affect the reliability and accuracy of participants’ responses [70, 71]. There is also the potential for misinterpretation in the operationalization of questions; for example, broad and non-temporal questions (e.g., “have you *ever* had to turn clients away”, versus “have you turned clients away *post-decriminalization?”*) may have been subject to varying interpretation by respondents. Furthermore, as the survey was administered after the implementation of the decriminalization policy, it is challenging to isolate the specific impacts of decriminalization from other potentially confounding factors, such as concurrent policies (e.g. prescribed pharmaceutical alternatives, municipal street sweeps), investments, and ecological factors (e.g., current drug supply). In addition, the survey design and the aggregate nature of the results further limit the ability to evaluate individual-or service-level site changes. For instance, we were unable to assess whether individual HR sites or specific services within these sites experienced unique impacts related to decriminalization, such as changes in funding (i.e., if specific programs offered within sites received additional funding or whether the funding was provided to the whole organization) or variations in client uptake that may not be applicable to other sites or services.

Future research should investigate the potential differences in site operations and utilization between sites in urban and rural locations, as well as explore the varying impacts of decriminalization across jurisdictions and on the different HR services offered within sites. Additionally, research should examine the impact of decriminalization on youth-specific sites, which were excluded from this study but may have experienced differential impacts. Other avenues worth exploring include the increase in demand on staff and resources, as well as the extent to which site operating budgets for HR programming may have changed since decriminalization. This study represents the first phase of a multi-year, longitudinal evaluation of BC’s decriminalization policy (see [72] for full methodology). Annual surveys will continue to assess impacts on HR services, allowing for the tracking of changes over time, and the inclusion of more robust quantitative analyses in future waves. While current findings are descriptive, aiming to establish a province-wide baseline, future waves will incorporate additional statistical approaches. Follow-up qualitative interviews with HR site representatives have also been conducted to explore site operations and policy implementation in greater depth. Complementary studies include analyses of the effects of decriminalization on OAT services [73], and an interrupted time series analysis assessing the impacts of decriminalization on several outcomes, including HR and health service utilization. Together, these efforts aim to provide a comprehensive understanding of decriminalization’s impacts on service operations and utilization.

## Conclusion

The findings from this study highlight several key challenges and opportunities for HR sites in BC in the context of decriminalization. While many HR sites reported increased client engagement following the policy’s implementation, they continue to face persistent barriers, such as limited capacity, resource constraints and insufficient funding to meet the rising demand. A lack of staff training on decriminalization has further strained HR sites’ ability to effectively support PWUD. Additionally, the continued presence of police activity near HR sites remains a concern, as it can perpetuate stigma and deter service utilization. Moreover, the limited availability of supervised inhalation services, despite inhalation being the most common route of administration associated with drug toxicity deaths, represents a critical gap in harm reduction efforts.

While our findings are based on self-reported, site-level data, and may not be generalizable to all settings, they nonetheless identify actionable issues that warrant immediate policy attention. Addressing these issues will require targeted, needs-based investments to expand HR services, increase staffing capacity, and improve infrastructure to respond to current and future demand. Province-wide, standardized training on decriminalization, tailored to both frontline and management staff, is also essential to support implementation, and ensure that staff are equipped to respond to evolving service needs. In parallel, cross-sectoral efforts to reduce visible police presence near HR sites are also necessary to mitigate stigma and improve service access for PWUD.

Responsibility for these changes spans several key stakeholders. While BC’s provincial government plays a central role in policy oversight and funding, regional health authorities are crucial for service implementation and adaptation. Other essential actors include the municipal governments, law enforcement agencies, community organizations, and service providers. Coordinated, multi-level collaboration is essential to align policy goals with the operational realities and capacities of HR service delivery. Ultimately, the long-term success of BC’s decriminalization policy hinges on its integration within a well-resourced, equitable, and responsive system of care. Strengthening service infrastructure, enhancing workforce readiness, and removing structural barriers will be essential to ensuring that HR sites can function as low-barrier, effective entry points to care and support for PWUD. Without these complementary efforts, the intended health and social benefits of decriminalization may not be fully realized, leaving critical gaps in care for PWUD.

## Data Availability

No datasets were generated or analysed during the current study.

## Abbreviations

BC: British Columbia
BCCDC: BC Centre for Disease Control
HR: Harm Reduction
IQR: Interquartile Range
MHSU: Mental Health and Substance Use
OAT: Opioid Agonist Treatment
OPS: Overdose Prevention Sites
PWUD: People Who Use Drugs
RAAC: Rapid Access Addiction Clinics
RAAM: Rapid Access to Addiction Medicine
REDCap: Research Electronic Data Capture
RI: Index of Remoteness
SCS: Supervised Consumption Sites
SD: Standard Deviation
